# Transcriptomic responses to wounding: meta-analysis of gene expression microarray data

**DOI:** 10.1186/s12864-017-4202-8

**Published:** 2017-11-07

**Authors:** Piotr Andrzej Sass, Michał Dąbrowski, Agata Charzyńska, Paweł Sachadyn

**Affiliations:** 1 Department Molecular Biotechnology and Microbiology, Gdańsk University of Technology, Gdańsk, Poland; 2 Laboratory of Bioinformatics, Neurobiology Center, Nencki Institute of Experimental Biology of Polish Academy of Sciences, Warsaw, Poland

**Keywords:** Wound healing, Wound repair, Tissue injury; regeneration, Gene expression microarray, Transcriptomics

## Abstract

**Background:**

A vast amount of microarray data on transcriptomic response to injury has been collected so far. We designed the analysis in order to identify the genes displaying significant changes in expression after wounding in different organisms and tissues. This meta-analysis is the first study to compare gene expression profiles in response to wounding in as different tissues as heart, liver, skin, bones, and spinal cord, and species, including rat, mouse and human.

**Results:**

We collected available microarray transcriptomic profiles obtained from different tissue injury experiments and selected the genes showing a minimum twofold change in expression in response to wounding in prevailing number of experiments for each of five wound healing stages we distinguished: haemostasis & early inflammation, inflammation, early repair, late repair and remodelling. During the initial phases after wounding, haemostasis & early inflammation and inflammation, the transcriptomic responses showed little consistency between different tissues and experiments. For the later phases, wound repair and remodelling, we identified a number of genes displaying similar transcriptional responses in all examined tissues. As revealed by ontological analyses, activation of certain pathways was rather specific for selected phases of wound healing, such as e.g. responses to vitamin D pronounced during inflammation. Conversely, we observed induction of genes encoding inflammatory agents and extracellular matrix proteins in all wound healing phases. Further, we selected several genes differentially upregulated throughout different stages of wound response, including established factors of wound healing in addition to those previously unreported  in this context such as *PTPRC* and *AQP4*.

**Conclusions:**

We found that transcriptomic responses to wounding showed similar traits in a diverse selection of tissues including skin, muscles, internal organs and nervous system. Notably, we distinguished transcriptional induction of inflammatory genes not only in the initial response to wounding, but also later, during wound repair and tissue remodelling.

**Electronic supplementary material:**

The online version of this article (10.1186/s12864-017-4202-8) contains supplementary material, which is available to authorized users.

## Background

Wound healing is a complex phenomenon during which the integrity of damaged tissue is restored. Wound response and healing are dynamic processes, where four main phases are distinguished: haemostasis lasting from seconds to about one hour, inflammation lasting from hours to days, followed by repair and remodelling, lasting from days to weeks and weeks to months, respectively [1]. The lengths of these phases depend on tissue type but they also show individual variance. The process involves not only the proliferation and differentiation of residual cells, but also cell migration, and apoptosis, as well as the formation and degradation of extracellular matrix (ECM). The course of response to injury and wound repair depends on tissue and injury type. The variety of cell types found in the wound area can be depicted using the example of full-thickness skin wound. In this type of wounding, platelets, which come first to the injury site in order to stop bleeding, are acellular factors, but they contain mRNA and display translational activity [2]. The residual mast cells, as well as incoming neutrophils and macrophages, contribute to the inflammatory phase. Neutrophils, typically arrive within 24 h after injury; they eliminate infectious agents and die out by apoptosis on the third day after injury to be replaced by incoming monocytes which differentiate into macrophages. Macrophages digest necrotic debris and other foreign bodies. Generally, the resolution of inflammation occurs within a week following injury, but macrophages in the wound area may remain for as long as months. Dermal fibroblasts and keratinocytes play main roles in the repair phase. The first produce extracellular matrix which forms the scaffolds in the place of lost tissue, the latter severe from wound edges, migrate and proliferate, thus re-epithelizing the wound. Activated fibroblasts proliferate and migrate into the injury site within 3-4 days after injury. Vascularization of extracellular matrix, which is connected with proliferation of endothelial cells, results in the formation of granulation tissue. Damaged peripheral neurons are degraded shortly after injury, proliferating Schwann cells form tubes providing conduits for axonal regeneration. The importance of nerve regeneration is not restricted to nervous system repair. Tissue regeneration is known to be nerve dependent [3]. Remodelling initiates a week after injury when fibroblasts differentiate into myofibroblasts. The phase may overlap with repair but it can last as long as years. Myofibroblasts produce α-smooth muscle actin and collagen. While smooth muscle actin filaments enable wound contraction, the deposition of collagen fibres leads to the formation of scar. Once the wound is contracted, myofibroblasts become quiescent and undergo apoptosis. Surface cutaneous injury, unlike the full-thickness one, is associated with little or no ECM production. Consequently, the repair is achieved by re-epithelization and no scarring occurs.

Whereas the processes of haemostasis and inflammation share similarities in different tissues, the phases of repair and remodelling display more differences dependent on tissue and injury type. Several organ specific key features of response to wounding are outlined below. The injuries of CNS are characterized by limited infiltration of lymphocytes due to blood-brain barrier, the lack of axonal regeneration, the repair by glial scarring by astrocytes, and almost no remodelling. Bone repair is usually perfect and it is mediated by osteoclasts which digest the necrotic bone, chondrocytes responsible for the ECM production in the repair of large gaps, and osteoblasts producing unmineralised bone matrix in the repair of small gaps. Cardiomyocytes, as a rule, show no proliferative activity and fibrous scars are formed as the consequence of heart injuries [4]. Quite the opposite, hepatocytes proliferate after liver injury and complete restoration of the organ has been reported after two-thirds liver resection within a week [5].

Though scar formation is a typical outcome of wound healing in mammals, unusual wound repair phenomena are observed in foetal and neonatal periods. Scarless skin wound healing occurs in mammals in the foetal period up to the beginning of the 3rd trimester [6–8]. On the other hand, burn wounds result in scarring even in the foetus [9]. The distinguishing features of scarless wound healing are weaker inflammatory response, decreased collagen deposition, and an increase in matrix metalloproteinase activity [10]. In adult mammals, the repair of heart injuries is connected with the formation of fibrous scars albeit the hearts of murine neonates up to day 5 have been reported to heal perfectly [11, 12].

Some strains and species, even among mammals, display impressive regenerative abilities at adulthood. Perfect skin wound healing has been reported in the spiny mouse [13] and the “Nude”, Foxn1 deficient mouse [14].

The processes of wound response and repair involve multiple factors and are dependent on multiple genes. This is why the application of genomic analyses offers great perspectives and advantages. On the other hand, its dynamic nature makes wound response difficult to characterize in a single experiment. Another complication is associated with delimiting the border between the normal and injured tissue in order to excise precisely the tissue in the wound area.

A number of gene expression microarray experiments in the tissues collected from the wound area have been carried out in recent years. The idea of this study is to investigate for common, remarkable transcriptomic responses to wound in different tissues and species.

## Methods

### Microarray data

We downloaded gene expression datasets from Gene Expression Omnibus database (www.ncbi.nlm.nih.gov/gds). We excluded from the analysis the datasets which did not include uninjured tissue as a control as well as those obtained from cultured cells from the wound area. We replaced negative signal values that correspond to absent transcripts and rarely occur in a few datasets, with 1.000, the value belonging to the lowest percentiles. Before further calculations, we back-transformed the gene expression signal values given as logarithms in the original datasets. The information on the analysed microarray datasets is summarized in Additional file 1.

### Algorithm of analysis

We averaged gene expression signals for biological replicates and determined the changes in expression for each transcript by dividing the mean gene expression signal in the wounded tissue to that of the control uninjured tissues (Additional file 1). In the case where a transcript was represented by more than a single probe set, we chose the highest fold change obtained for this transcript. We excluded all probe sets that were annotated to multiple transcripts. In order to allow cross species comparisons, we translated mouse and rat orthologues to human counterparts with OrthoRetriever (https://lighthouse.ucsf.edu/orthoretriever/).

We assembled the ratios representing gene expression fold changes in a single table and excluded from further analysis the transcripts that were not present in at least a half of the analysed series. We divided the data into five groups representing the sequence of wound response phases: haemostasis & early inflammation (0-24 h), inflammation (1-3 days), early wound repair (3-7 days), late wound repair and remodelling (7-14 days), remodelling (more than 14 days). We showed in the results but we did not include in the calculations three samples representing the tissues collected immediately after injury (time zero).

**Fig. 1:**
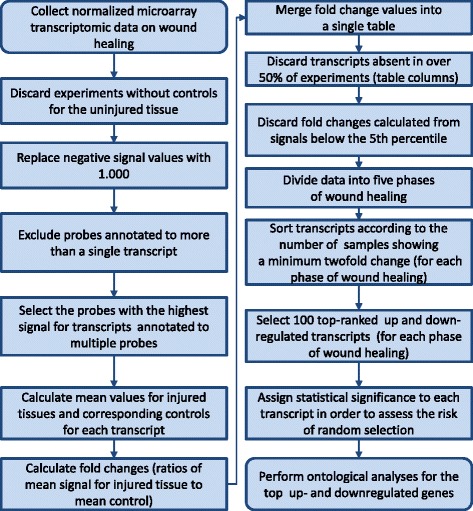
Data analysis pipeline

We ranked the transcripts according to the percentage of samples with the expression fold changes higher than or equal to 2.0 so as to sort out the genes induced in response to wounding in the majority of samples. We performed an analogical sorting, according to the percentage of samples with expression fold changes lower than or equal to 0.5 to single out the genes repressed in response to wounding. For further analysis, we selected one hundred top-ranked up- and one hundred top-ranked downregulated genes. Figure 1 depicts the path of data analysis.

### Statistical analysis

As the statistics to rank the genes, separately for each phase of the wound response and separately for the up- and downregulated genes, we used the number of the treated samples, in which a particular gene was regulated more than twofold, i.e. its fold change was >2.0 or <0.5, relative to the average expression of this gene in the control samples from the same experiment.

To assign a *p*-value to a gene from the top one hundred of upregulated or the top one hundred of downregulated genes, we set the null-hypothesis that the fold changes of target genes came from the same distribution as the fold changes of the control samples (i.e. the H0 states: there is no difference between the treatment and the control samples). To obtain the empirical distribution of fold changes in control samples for a given phase, we used the data from all the experiments pooled together as a single distribution. From this distribution we estimated the probability (as the observed frequency) that the fold change for a given gene is greater than 2.0 (for the upregulated genes) and separately, that the fold change is less than 0.5 (for the downregulated genes). The probabilities were then used as the probability of a success in Bernoulli trials associated with the binomial distributions for target genes. For each gene the binomial distribution was tailored separately with the number of trials equal to the number of treated samples in each phase and the number of success defined as the number of treated samples that was regulated more than twofold. In correspondence to the binomial distributions we calculated the one-side *p*-values i.e. the probabilities that a given gene was regulated more than twofold in the observed treated samples under the null hypothesis.

**Fig. 2:**
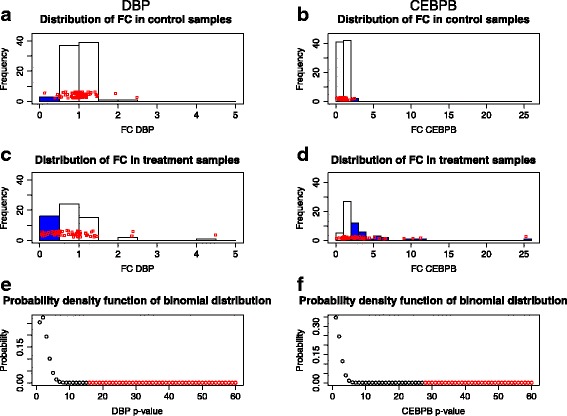
Diagrams depicting the procedure of *p*-value assigning to transcripts differentially regulated in response to wounding. **a** The histogram of the *DBP* transcript standardized fold changes in the control samples pooled together for all experiments in the haemostasis phase. Blue bar denotes the number of samples with fold change (FC) less than 0.5. **b** The histogram of the *CEBPB* transcript standardized FC in the control samples pooled together for all experiments in the haemostasis phase. Blue bar denotes the number of samples with FC greater than 2.0. **c** The histogram of the *DBP* transcript standardized fold changes in the treatment samples pooled together for all experiments in the haemostasis phase. Blue bar denotes the number of samples with FC less than 0.5. **d** The histogram of the *CEBPB* transcript standardized fold changes in the treatment samples pooled together for all experiments in the haemostasis phase. Blue bars denote the samples with FC greater than 2. **e** The theoretical binomial distribution of the *DBP* transcript standardized FC in the treatment samples under the null hypothesis. The red dots denote the *p*-value corresponding to the number of treatment sample with FC less than 0.5. **f** The theoretical binomial distribution of the *CEBPB* transcript standardized FC in the treatment samples under the null hypothesis. The red dots denote the p-value corresponding to the number of treatment sample with FC greater than 2.0

**Fig. 3:**
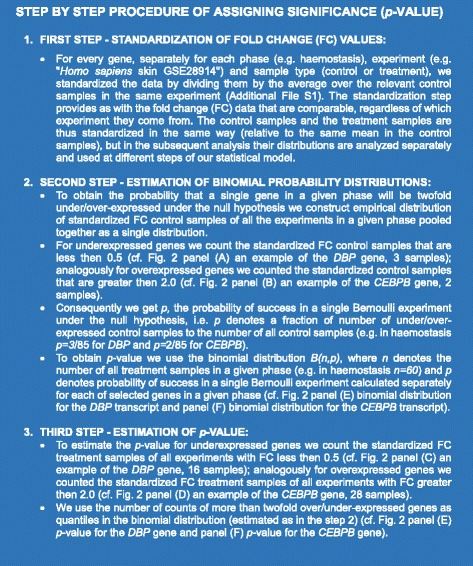
Step by step procedure of assigning significance (p-value) to a specific transcript differentially regulated in response to wounding

A step by step example of the procedure for *p*-value assigning to a specific transcript is described in Figs. 2 and 3.

For some genes, the fold change was less than twofold in all the control samples, relative to the average expression over all the control samples. In such cases the observed frequency of success in the Bernoulli trial, and consequently the binomial *p*-value under our model would be zero. To provide an upper bound of the *p*-values in such cases, we used one over the number of the control samples for a given phase as the probability of success. Those genes selected as upregulated or downregulated that did not prove significant after statistical evaluation (p-value >0.05) were excluded from further analysis.

### Gene ontology analysis

We conducted the functional analyses of differentially expressed genes using DAVID 6.8 provided by National Institute of Allergy and Infectious Diseases (NIAID) [15] with the default set of background genes for *Homo sapiens.* The statistical significance of gene sets enrichments were determined with Fisher’s exact test, which is an integral procedure of this bioinformatics tool.

## Results and discussion

### Identification of genes induced and repressed in response to injury

In this meta-analysis we attempted to assemble the greater part of transcriptomic data on gene expression in response to wounding. We compared the available transcriptomic profiles of injured tissues in the search of the traits shared by different wound models. The analysed gene expression profiles represent diverse tissues including heart, liver, skin, bones, epithelia, brain, spinal cord and nerves from a few species (rat, mouse, human), and different stages of wound response as well as injury types.

The datasets we assembled were divided into five groups corresponding to the sequence of wound response phases: haemostasis & early inflammation, inflammation, early wound repair, late wound repair, and remodelling. We ranked the transcripts in each group according to the percentage of samples displaying a twofold change in expression to sort out top 100 up- and top 100 downregulated genes (Additional file 2).

### Common traits of transcriptomic responses to wound in different tissues

**Fig. 4:**
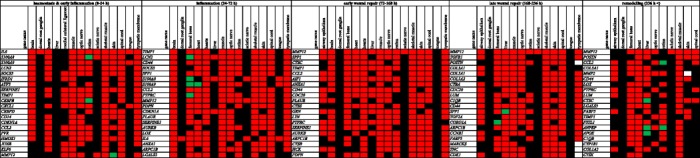
Twenty top-ranked upregulated genes representative of different wound healing phases. Red fields indicate at least a twofold upregulation in the prevailing part of experiments. Green fields indicate at least a twofold downregulation in the prevailing part of  experiments. Black fields indicate no substantial changes in gene expression and blank fields indicate no data

**Fig. 5:**
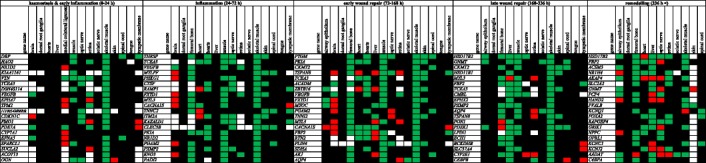
Twenty top-ranked downregulated genes representative of different wound healing phases. Red fields indicate at least a twofold upregulation in the prevailing part of experiments. Green fields indicate at least a twofold downregulation in the prevailing part of experiments. Black fields indicate no substantial changes in gene expression and blank fields indicate no data

Despite the diversity of analysed tissues, we found a number of transcripts displaying similar responses to injury in the major part of samples within the distinguished phases. In order to show the representative examples of transcriptional responses to wounding, distinctive of each tissue, we prepared lists of 20 top-ranked genes induced and repressed in each phase (Figs. 4 and 5). In this synthetic view, the genes that displayed at least a twofold change in expression in a greater number of experiments for a given tissue are indicated as either up- or downregulated. The levels of most transcripts analysed from 4 to 24 h showed neither significant increase nor decrease, as it is shown by the heat map of expression fold changes (Figs. 4 and 5, Additional file 2). For gene expression profiles in the initial phases of healing: haemostasis &early inflammation and inflammation, we revealed disparate transcriptomic responses to wound. In the later phases of repair and remodelling, a number of genes showed similar expression profiles in different tissues  (Figs. 4 and 5).

### Gene ontology analysis

**Fig. 6:**
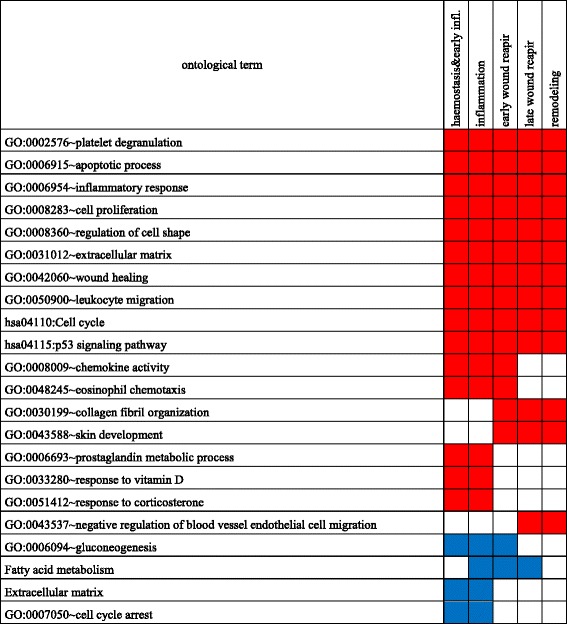
Ontological categories associated with the genes differentially regulated in different phases of wound response. The gene set enrichment analyses were performed for the top 100 up- and the top 100 downregulated genes for each phase of wound response. The ontological terms were statistically significant as determined by Fisher’s exact test and Benjamini correction for multiple comparisons (*p*-value adjusted <0.05)

In order to explore the functional associations of genes displaying changes in expression after wounding, we employed gene set enrichment analysis. The results of the analysis are summarized in Fig. 6. We found an enrichment of upregulated genes associated with inflammatory response, chemotaxis of monocyte macrophage and neutrophils, cell proliferation and apoptosis for all phases of response to wounding, while of those related to chemokine activity and eosinophil migration only until early wound repair. In the haemostasis and inflammation phases, we revealed induction of genes involved in response to vitamin D and corticosterone. The later phases of wound healing showed increased expression of genes responsible for collagen fibril synthesis and organisation as well as skin development.

An analogical analysis conducted for downregulated transcripts exposed an enrichment of downregulated genes involved in carbohydrate and lipid metabolism with the exception for the remodelling phase. Early phases of response to wounding showed an overrepresentation of downregulated genes responsible for cell cycle arrest.

### Genes differentially regulated along different wound healing phases

**Fig. 7:**
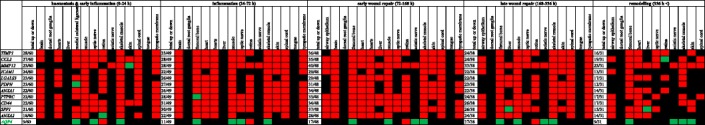
The genes upregulated along different phases of wound response and healing in different tissues. Red fields indicate at least a twofold upregulation in the prevailing part of experiments. Green fields indicate at least a twofold downregulation in the prevailing part of experiments. Black fields indicate no substantial changes in gene expression and blank fields indicate no data

The transcriptomic response to wounding is highly variable dependent on injured tissue, the type of injury and the time following injury. However, we were able to distinguish the genes differentially regulated in the majority of analysed datasets in different phases of wound response in different tissues. We singled out 11 genes upregulated and 1 downregulated through the whole process of wound healing. The expression patterns of those genes are presented in Fig. 7 and their functions are summarized in Table 1. This set of genes is significantly enriched for those associated with extracellular matrix organisation and disassembly, inflammatory response and chemotactic activity (Table 2).

**Table 1:** Functions of genes upregulated along different phases of wound response and healing in different tissues

Gene symbol	Gene name	Function of genes product
*ANXA1 ↑*	Annexin A1	membrane- phospholipid binding protein which has anti-inflammatory activity
*ANXA2 ↑*	Annexin A2	autocrine factor which enhances osteoclast formation and bone resorption
*CCL2 ↑*	C-c motif chemokine ligand 2	factor augmenting anti-tumor activity of monocytes
*CD44 ↑*	CD44 molecule (Indian blood group)	receptor for hyaluronic acid interacting with osteopontin, collagens, and matrix metalloproteinases and involved in lymphocyte activation and homing, haematopoiesis, and metastasis
*ICAM1 ↑*	Intercellular Adhesion Molecule 1	cell surface glycoprotein expressed on endothelial and immune system cells
*LGALS3 ↑*	Galectin 3	plays a role in numerous cellular functions including apoptosis, innate immunity, cell adhesion and T-cell regulation; exhibits antimicrobial activity
*MMP12 ↑*	Matrix metallopeptidase 12 (macrophage elastase)	degrades elastin; involved in extracellular matrix breakdown in development, remodeling but also in aneurysm, metastasis
*PDPN ↑*	Podoplanin	integral membrane glycoprotein proposed as a marker of lung injury
PTPRC ↑ *	Protein Tyrosine Phosphatase, Receptor Type C	essential regulator of T- and B-cell antigen receptor signaling; suppresses JAK kinases, thus functioning as a regulator of cytokine receptor signaling
*SPP1 ↑*	Secreted Phosphoprotein 1	involved in the attachment of osteoclasts to the mineralized bone matrix; a cytokine upregulating expression of interferon-gamma and interleukin-12
*TIMP1* ↑	TIMP metallopeptidase inhibitor 1	inhibits most known matrix metalloproteinases; promotes cell proliferation in a wide range of cell types
AQP4 ↓*	Aquaporin 4	water-selective channel in the plasma membrane; a predominant aquaporin found in brain

The information on gene functions was derived from the data collected in the GeneCards database (www.genecards.org), which includes (Entrez Gene summary and UniProtKB/Swiss-Prot:Function)

The downregulated gene is marked with a downwards arrow↓, the upregulated genes are indicated with upwards arrows↑.

The genes which have not been reported so far among the main players in wound response are distinguished with asterisks

**Table 2:** Functional annotation for 12 genes differentially regulated throughout all phases of wound response

Term	Number of genes	*p*-value	Genes
GO:0022617~extracellular matrix disassembly	4	**1.43E-05**	*CD44, MMP12, SPP1, TIMP1*
GO:0005615~extracellular space	7	**5.39E-05**	*ICAM1, CCL2, LGALS3, ANXA1, SPP1, ANXA2, TIMP1*
GO:0008360~regulation of cell shape	4	**8.91E-05**	*ICAM1, CCL2, PDPN, ANXA1*
GO:0009986~cell surface	3	**2.16E-04**	*CCL2, LGALS3, ANXA1*
GO:0002548~monocyte chemotaxis	3	**3.31E-04**	*ICAM1, CCL2, CD44*
GO:0070374~positive regulation of ERK1 and ERK2 cascade	3	5.58E-03	*ICAM1, CD44, SPP1*
GO:0030198~extracellular matrix organization	5	6.96E-03	*ICAM1, PTPRC, CD44, ANXA1, ANXA2*
GO:0048246~macrophage chemotaxis	2	8.49E-03	*CCL2, LGALS3*
GO:0006954~inflammatory response	3	2.44E-02	*CCL2, ANXA1, SPP1*
GO:0043434~response to peptide hormone	2	2.85E-02	*ANXA1, TIMP1*
GO:0043066~negative regulation of apoptotic process	3	3.43E-02	*CD44, ANXA1, TIMP1*
GO:0030593~neutrophil chemotaxis	2	4.24E-02	*CCL2, LGALS3*

The *p*-values for the terms significant after Benjamini correction (*p*-value adjusted <0.05) are distinguished with bold font

The 12 genes differentially regulated during all wound healing phases are listed in Fig. 7

Genes induced by wounding, such as *SERPINA3* could be predicted to improve wound repair. Indeed, topical administration of Serpina3 rescues impaired wound healing in diabetic mice [16]. If a gene is involved in wound response, the deficiency (knockout) of this gene is expected to entail an impaired wound healing. This occurs in the case of a few upregulated genes we distinguished in this study such as *CCL2 and LGALS3*. The knockout of the *Ccl2* gene in mice leads to impaired re-epithelisation and angiogenesis in skin wounds [17]. The *Lgals3* knockout was shown to reduce the re-epithelisation rate in murine skin, but the overall wound healing rate remained largely unaffected. Nevertheless, it might be an important factor in chronic wound development [18]. However, there are animal models where positive impacts of single gene deletion have been shown. Improved or accelerated wound healing have been reported in the case of a few other genes upregulated during different wound healing phases as found in this analysis (Fig. 7), *Cd44* and *Mmp12*. The *Cd44* deficiency was reported to improve tendon healing [19]. Also the deficiency of *Mmp12* was found to have some positive effects on wound repair [20]. We could list a number more of knockout models reported to show either accelerated or improved wound healing including *Hif1a* [21]*, Hoxb13* [22]*, Ifng* [23]*, Il10* [24]*, Sparc* [25]*, Tgfbr2* [26]*, Il1r1* [27]*,* and *Tnfrsf1a* [28]. The mechanisms of healing were developed in the course of evolution for rapid decontamination and closing wounds. The examples of improved or accelerated healing in the knockout models indicate that response to wounding could be modified in a number of ways so as to be directed into regenerative repair.

The group of 12 transcripts which display the changes in expression levels throughout different stages of wound response and repair include mainly the genes of established roles in this process. However, two of them, *PTPRC* and *AQP4* have not been described as important factors associated with wound response or wound healing and these genes are worth considering as candidates for further wound repair and regeneration studies.

### Critical remarks

It should be noted that the examination of wound response using microarray techniques is connected with several fundamental problems. **The excision of wound area** - it is difficult to obtain sufficient amounts of tissues and to excise precisely the injured tissue without collecting the neighbouring normal tissues. **Dynamics of the response to injury and wound repair** - the available microarray profiling methods allow the examination of several time points, but not continuous tracking of the process. In addition, the transcriptomic profiles reflect not only the alterations in gene expression levels but also the changing content of cell types resulting from cell proliferation, cell death and the accumulation of incoming cells. **The limitations of microarray profiling -** most expression microarrays do not include a substantial part of known genes, in particular the miRNA genes. **The regulation on the translational level** – a substantial proportion of genes are regulated on the posttranscriptional level [29].

The **interpretation of microarray results** adds another complication. Different probe sets for the same gene may produce dramatically different signals. Such different signals may reflect either the presence of two splicing variants or incomplete synthesis of cDNA of long transcripts. In this study, we assumed the algorithm selecting these probe sets for which the highest changes in gene expression were determined.

We would like to stress that the fold changes given here are not supposed to show the accurate expression levels but to visualize trends rather. The exact determination of expression change for a specific gene requires careful validation with alternative reference methods and splice variants should be considered in such examination. This is why we focused the analysis on overall tendencies. It seems that the approach we assumed, was factual, as our analysis indicated a number of genes, which with no doubt are associated with wound response.

The **experimental conditions are not standardized**, which could be considered as a weakness of this meta-analysis. On the other hand, this diversity of experimental conditions eliminates, in some way, potential batch effects, thus facilitating the search for common traits in wound response.

## Conclusions

A number of gene expression microarray studies of wound response are publicly available. This study is, to our knowledge, the first meta-analysis which compares a selection of available gene expression profiles obtained from the tissues collected from the area of injury. The data used in this study represent several experiments which were different regarding the microarray used, the set probes, and the time points after injury. This variation complicates the comparison of gene expression levels, but on the other hand, it offers a certain type of advantage - the observations are not restricted to a single type of experiment and are verified in different experimental conditions. Though no gene was found to be induced/repressed for all tissues, time points, and injury types, the analysis revealed a group of genes (Fig. 7) that change their expression after wounding in the majority of analysed samples along different healing phases, thus exposing certain common features of transcriptomic response to wounding. Among the genes showing transcriptional responses to wound along different phases, we found those of known roles in this process such as *CD44, MMP12, ICAM1* as well as those which have not been listed among the main players involved in wound repair such as e.g. *PTPRC* and *AQP4*. While the transcriptomic responses to injury showed little consistency in the initial phases of haemostasis and inflammation, we found that there are induced and repressed genes characteristic of the subsequent healing and remodelling phases shared by the diverse selection of analysed tissues. It is also worth noting that different groups of immune response genes were activated not only in the inflammatory phase of wound response, but also during repair and remodelling.

## Additional files

Additional file 1: The datasets included in the analysis. (XLSX 24 kb)

Additional file 2: Genes up- and downregulated in different wound healing phases. (XLSX 660 kb)

## Abbreviations

CNS: central nervous system
ECM: extracellular matrix
